# Valorization of Metallurgical Slags into High-Performance Lithium Ferrite for Efficient CO_2_ Capture

**DOI:** 10.3390/molecules31091457

**Published:** 2026-04-28

**Authors:** Amelia Jiménez-Alcántara, Carlota García-González, Rosa-María Ramírez Zamora, Brenda Alcántar-Vázquez

**Affiliations:** 1 Instituto de Ciencias de la Atmósfera y Cambio Climático, Universidad Nacional Autónoma de México, Mexico City 04510, Mexico; ameliajimenezalc@comunidad.unam.mx; 2 Instituto de Ingeniería, Universidad Nacional Autónoma de México, Avenida Universidad 3000, Coyoacán, Mexico City 04510, Mexico; cgarciag@iingen.unam.mx (C.G.-G.);

**Keywords:** metallurgical slag, waste valorization, CO_2_ capture, lithium ferrite, CO_2_ sorbent

## Abstract

Copper slag was used as a raw material to prepare lithium ferrite by the solid-state reaction method at different Li:Fe molar ratios. The obtained materials were characterized by XRD, SEM, and N_2_ adsorption–desorption, and their CO_2_ capture behavior was evaluated using thermogravimetric and temperature-programmed techniques. A 7:1 Li:Fe molar ratio allowed to obtain Li_5_FeO_4_, as well as Li_4_SiO_4_, due to the high silicon content in the slag. CO_2_ sorption tests showed that, as temperature increases, CO_2_ capture increases up to 675 °C. Slag-ferrite achieved a maximum CO_2_ capture of 20 wt% at 675 °C (*P_CO_*_2_ = 0.2), equivalent to 62.5% of the CO_2_ sorption of reagent-grade ferrite (32 wt%). Kinetic analysis of CO_2_ capture using the Avrami–Erofeev model indicated that bulk diffusion is the rate-controlling step. These results provide quantitative evidence on the use of copper slag in the preparation of lithium ferrites, with potential application in a high-temperature CO_2_ capture process.

## 1. Introduction

Carbon dioxide (CO_2_) is a greenhouse gas emitted by natural processes, such as respiration and volcanic eruptions, as well as by anthropogenic activities, such as deforestation, land-use changes, and the combustion of fossil fuels. In 2023, global anthropogenic CO_2_ emissions were estimated at 40.6 Gt [1]. The increasing concentration of CO_2_ in the atmosphere has prompted extensive efforts to develop management strategies. Among these, carbon capture, utilization, and storage (CCUS) technologies have emerged as promising solutions for industrial decarbonization and net CO_2_ removal [2]. A critical component for the effective deployment of CCUS is the CO_2_ sorbent material. CO_2_ sorbents are classified as high- and low-temperature materials. The former refers mostly to alkali metal oxides, alkali silicates, alkaline zirconates, and hydrotalcites. Low-temperature adsorbents include porous carbon-based materials, solid amine materials, metal–organic frameworks (MOFs), zeolites, and covalent organic frameworks (COFs) [2]. Such materials must exhibit high CO_2_ sorption capacity, stability under varying gas-stream conditions, favorable kinetics, regenerability, and low cost. These technologies are particularly relevant in industrial sectors with high CO_2_ emissions, such as cement manufacturing, steel production, oil refining, and petrochemical processes, where flue gases are generated at elevated temperatures [3].

Lithium- and sodium-based materials have been extensively studied for their superior performance as high-temperature CO_2_ sorbents (T ≥ 400 °C) [4,5]. Lithium-based ceramics, such as lithium cuprate (Li_2_CuO_2_), lithium aluminate (Li_5_AlO_4_), lithium orthosilicate (Li_4_SiO_4_), and lithium ferrites (Li_5_FeO_4_), have demonstrated remarkable CO_2_ capture capacities over broad temperature ranges (300–800 °C) [6]. Among these, ferrites have attracted growing attention due to their dual application in CO_2_ capture and H_2_ production [6,7,8,9,10,11,12]. Recent studies indicate that lithium ferrites, particularly Li_5_FeO_4_, exhibit high sorption capacities and thermal stability over a wide temperature range [13].

Li_5_FeO_4_, synthesized via solid-state reaction between lithium oxide and Fe (III) oxide, shows exceptional potential as a high-temperature CO_2_ sorbent, as evidenced by both theoretical and experimental investigations [6,11,14]. Theoretical models suggest that, when Li_5_FeO_4_ captures CO_2_, it releases almost twice as much heat as LiFeO_2_. Under post-combustion conditions (PCO_2_ = 0.1), LiFeO_2_ captures CO_2_ only below 192 °C, whereas Li_5_FeO_4_ sustains the carbonation process up to 700 °C. Consequently, Li_5_FeO_4_ can adsorb CO_2_ across a broad temperature range, producing Li_2_CO_3_ and LiFeO_2_ (reaction 1). In contrast, LiFeO_2_ is not thermally efficient for CO_2_ capture (reaction 2). Overall, Li_5_FeO_4_ demonstrates a CO_2_ capture capacity of 12.9 mmol per gram of material (567.2 mg/g_material_) [14].


(1) $${Li}_{5}FeO_{4}+2CO_{2}\to {2Li}_{2}CO_{3}+Li{FeO}_{2}$$

(2) $$2LiFeO_{2}+CO_{2}\;\to \;{Li}_{2}{CO}_{3}+{Fe}_{2}O_{3}$$

Within the framework of a circular economy, utilizing industrial waste as raw materials for synthesizing CO_2_ sorbents offers several notable advantages, including wide availability, low cost, high reactivity, and a complex matrix that can enhance capture and regeneration. Waste materials explored for this purpose include sludge, biomass, discarded polymers, fly ash, metallurgical slags, construction and demolition waste and other industrial by-products [15,16,17,18,19].

The use of metallurgical slags as raw materials for CO_2_ sorbents offers additional advantages, including their wide availability as a source of metal oxides, making them a replacement for commercial reagents [20,21]. For instance, Bejarano-Peña et al. used electric arc furnace slags to prepare Li_4_SiO_4_, achieving CO_2_ capture of 104.6 mg/g_material_ at 600 °C with 5 and 20 vol% CO_2_ after a simple addition of 20% K_2_CO_3_ [22]_._ Blast furnace slag (BF) was also used to prepare Li_4_SiO_4_ with high stability and CO_2_ capture of 134 mg/g_material_ at 650 °C [20]. The composition of the slag led to the formation of secondary phases (Li_2_CaSiO_4_, LiAlO_2_, and LiAlSiO_4_) that enhanced CO_2_ capture and enabled high regeneration during cyclic testing [21]. Similarly, copper slags have been used to synthesize sorbents whose main active phases are Li_5_FeO_4_ and Li_4_SiO_4_. The highest CO_2_ uptake was observed at 725 °C, reaching 420 mg/g_material_ at the end of isothermal sorption, which is close to its theoretical capacity of 567.2 mg/g_material_ [13].

World copper production in 2024 reached 23 million metric tons [1]. Chile is the world’s largest producer of this metal, followed by the Democratic Republic of the Congo, Peru, China, Indonesia, the United States, Russia, Australia, Kazakhstan, and Mexico [23]. Copper is Mexico’s fourth most-produced metal, with approximately 2.2 metric tons of copper slag generated per ton of copper produced [24,25]. Like other metallurgical slags, copper slag is mostly disposed of near smelting facilities, causing environmental and social harm. Its chemical composition and physical and mechanical properties [25,26,27] have enabled its use in H_2_ production, wastewater treatment, and the manufacture of cement, concrete, bricks, and ceramics [24,28,29,30,31,32].

The main components of copper slag are iron oxide (Fe_2_O_3_) and silicon dioxide (SiO_2_), which together constitute approximately three-quarters of the material [24], making copper slags an ideal candidate to replace commercial reagents in the synthesis of Li_5_FeO_4_ and providing opportunities to valorize industrial waste while contributing to CO_2_ mitigation. Therefore, this study explores the potential of copper slag-based lithium ferrites as high-temperature CO_2_ sorbents, aiming to reduce industrial waste and pollutant emissions from energy-intensive processes.

## 2. Experimental

### 2.1. Characterization of Copper Slag

The copper slag was sourced from a Mexican industry, and its major and trace elements were determined by X-ray fluorescence (XRF) using a Rigaku Primus II spectrometer. X-ray diffraction (XRD) was carried out with an EMPYREAN diffractometer equipped with a Ni filter (λ = 1.54056 Å), a copper tube with a fine cone, and a PIXcel 3D detector. The patterns were measured over a 2θ range of 10–80° using a high-resolution routine with a step scan of 0.003° and an integration time of 40 s per pass. On the other hand, as part of the microstructural characterization, the N_2_ adsorption–desorption isotherm was obtained using a Bel-Japan Minisorp II instrument (BEL Japan, Inc., Osaka, Japan) at 77 K by the multipoint technique (N_2_ from Praxair, grade 4.8) and the specific surface area was calculated by the BET method. Before analysis, the sample was pretreated overnight at 120 °C in a nitrogen flow (40 mL min^−1^). The microstructural characterization was completed using scanning electron microscopy (SEM); backscattered electron (BSE) images and elemental mapping were obtained with a JEOL JMS-7600F microscope (JEOL Ltd., Tokyo, Japan).

### 2.2. Lithium Ferrite Preparation and Characterization

Slag-derived lithium ferrites were prepared using the solid-state reaction method by mixing lithium oxide (Li_2_O, 99.8%, Sigma-Aldrich, St. Louis, MO, USA) and copper slag as an iron oxide source in a Li:Fe molar ratio of 4:1, 5:1, 7:1, and 9:1 (an excess of 10%mole was added due to the lithium sublimation). The mixtures were pressed into pellets and calcined at 850 °C for 20 h. For comparison, pentalithium ferrite (Li_5_FeO_4_) was also prepared from reagent-grade Fe_2_O_3_ (99.5%, Aldrich) using the same calcination conditions. All calcined materials were homogenized in an agate mortar, and the resulting crystalline phases were identified by XRD using an Empyrean diffractometer (Malvern Panalytical, Malvern, UK) with CuKα radiation and a PIXcel3D detector (Malvern Panalytical, UK). Moreover, microstructural characterization was performed as previously described.

### 2.3. CO_2_ Sorption Tests

The CO_2_ sorption behavior of the resultant lithium ferrites was evaluated by thermogravimetric (TG) and temperature-programmed techniques. Temperature-programmed sorption–desorption experiments were conducted using a Belcat B instrument (BEL Japan, Inc., Japan) equipped with a thermal conductivity detector (TCD), which recorded changes in CO_2_ concentration in the outlet gas stream. Before temperature-programmed tests, the samples were pretreated in N_2_ flow (60 mL/min) at 800 °C to eliminate any previous carbonation. In these experiments, 50 mg of sorbents were kept in contact with a 5% mol CO_2_ gas stream (He balance, Praxair, certified standard) and heated to 900 °C at a rate of 5 °C min^−1^. Moreover, thermogravimetric experiments were performed on a Labsys Evo TG analyzer (Setaram Instrumentation, Caluire-et-Cuire, France) using 20 mg of sample. The dynamic CO_2_ sorption performance was obtained by heating the samples from room temperature to 850 °C (5 °C min^−1^), varying the CO_2_ partial pressure (*P*_CO2_) between 0.2 and 0.05 (using N_2_ as the gas balance). The CO_2_ sorption over time was measured between 400 and 700 °C. In each test, the temperature was increased in N_2_ flow (60 mL min^−1^); subsequently, the flow was changed to the desired *P*_CO2_ for 180 min. Finally, to elucidate the carbonation products, the slag-lithium ferrites were analyzed by XRD after CO_2_ sorption.

## 3. Results and Discussion

### 3.1. Physicochemical Characterization of Copper Slag

The chemical composition of as-received copper slag is shown in Table 1 (major elements are reported as oxides). Iron oxide was the main component, at 59.66 wt%, followed by silicon oxide at 26.52 wt%. Aluminum and potassium oxides were also identified in small amounts (1–5 wt%). Other oxides, such as CaO, MgO, and Na_2_O, were found to have a percentage less than one. These results confirm the high iron content in the slag, making it suitable for synthesizing lithium ferrite. In addition to major elements, traces of Cu, Zn, Mn, Cr, Ni and Zr, among others, were identified and quantified. The presence of these elements is consistent with the copper refining process.

**Figure 1:**
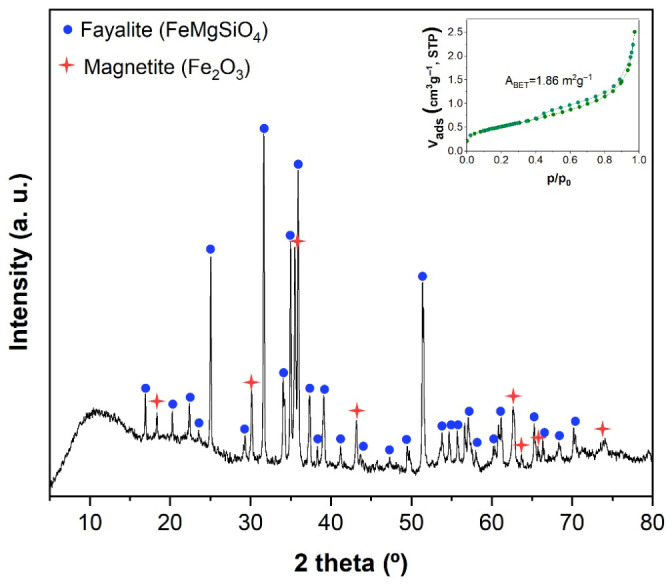
XRD pattern and N_2_ adsorption–desorption isotherm (inset) of copper slag.

**Figure 2:**
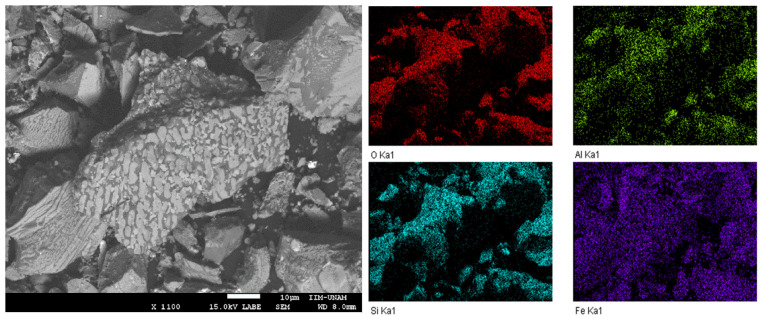
Scanning electron images and EDS elemental mapping of copper slag.

The XRD pattern of the slag (Figure 1) shows an amorphous halo at low 2-theta and characteristic peaks of fayalite (FeMgSiO_4_, ICSD 01 071 1667) and magnetite (Fe_2_O_3_, ICDD 98 003 5000). The abundance of each crystalline phase was determined by semiquantitative analysis using the RIR method. Fayalite was the most abundant phase in the copper slag with 67 wt%, and the remaining 33 wt% corresponds to magnetite. Then, the textural characterization was performed using N_2_ adsorption–desorption and SEM. The inset in Figure 1 shows the N_2_ adsorption–desorption isotherm, which, according to the IUPAC classification, is type II with an H3 hysteresis loop, characteristic of nonporous solids [33]. The surface area determined by the BET method was 1.86 m^2^g^−1^. The low-angle backscattered electron (LABE) image of copper slag (Figure 2) shows large polyhedral particles of around 1–70 μm. The presence of an amorphous phase over the particles was clear. The EDS mapping of the sample shows that O, Al, Si, and Fe are present with a highly homogeneous distribution on the surface of this slag.

### 3.2. Characterization of Lithium Ferrites

**Figure 3:**
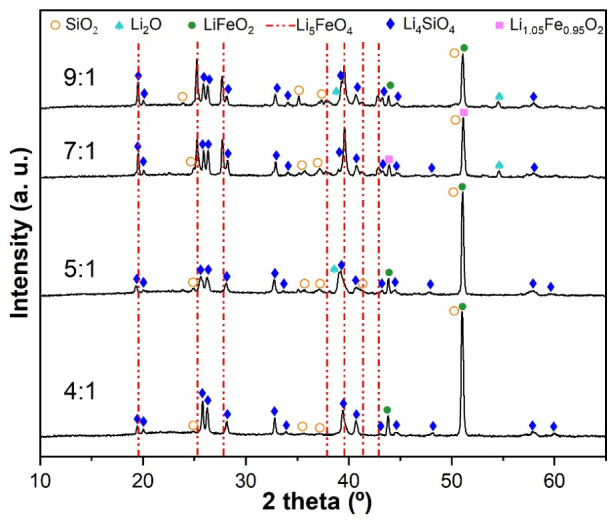
XRD patterns of the synthesis products with different Li:Fe molar ratios. Red dotted lines correspond to Li_5_FeO_4_ (PDF # 00-037-1151).

The obtained materials with different Li:Fe molar ratios were analyzed by XRD, as shown in Figure 3. The lithium oxide reacts first with the silica present in the slag to form Li_4_SiO_4_ due to the Gibbs free energy change for the reaction between Li_2_O and SiO_2_ to form Li_4_SiO_4_ (−207.62 kJ mol^−1^) being greater than that of the reaction between Li_2_O and Fe_2_O_3_ to obtain Li_5_FeO_4_ (−62.76 kJ mol^−1^) [11]. Therefore, Li_4_SiO_4_ (01-076-1085 PDF file) was identified in all Li:Fe molar ratios used. Moreover, low-lithium-content ferrite LiFeO_2_ (ΔG= −123.47 kJ mol^−1^), fitting with the 01-074-2284 PDF file, was formed before Li_5_FeO_4_ with the small amount of lithium available. Li_5_FeO_4_ was not obtained at 4:1 and 5:1 Li:Fe stoichiometric ratios; a higher Li:Fe ratio was required. At 7:1 and 9:1 Li:Fe molar ratios, Li_5_FeO_4_ (00-037-1151 PDF file) was identified. Li_2_O (01-080-4680 PDF file) was also observed in the XRD patterns, because, at high lithium content, some Li_2_O did not react.

**Figure 4:**
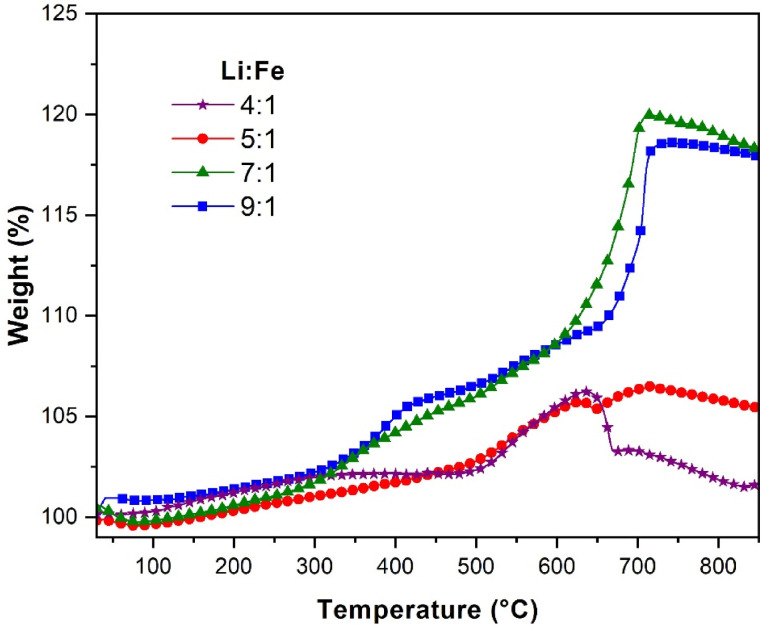
Dynamic TG curves of the slag-Li_5_FeO_4_ materials prepared using different Li:Fe molar ratios, *P*_CO2_ = 0.2.

Dynamic TG experiments with *P*_CO2_ of 0.2 were conducted to determine which material to continue working with: 7:1 or 9:1 (4:1 and 5:1 materials were also tested for comparison). As shown in Figure 4, the materials obtained from low lithium molar ratios (4:1 and 5:1) showed small CO_2_ capture above 500 °C, 5–6 wt%, consistent with the poor CO_2_ capture performance reported for Li_4_SiO_4_ at high temperature and low CO_2_ partial pressure [18,20,33,34,35]. Then, above 650 °C, the adsorption–desorption equilibrium changes, and desorption starts on the 4:1 material, where Li_4_SiO_4_ is the main crystalline phase. The desorption process in Li_4_SiO_4_ involves Li_2_CO_3_ and Li_2_SiO_3_, formed during the sorption stage, reacting to regenerate Li_4_SiO_4_ and release CO_2_ [5,34,35]. Whereas the 5:1 material shows inflection but no clear desorption, CO_2_ capture continues up to 850 °C; this behavior could be due to the presence of Li_2_O. These two materials contain LiFeO_2_; however, according to the literature, it can adsorb only a very low amount of CO_2_ (<2 wt%) between 200 and 450 °C [36,37]. Completely different curves were observed in the materials prepared with 7:1 and 9:1 Li:Fe molar ratios, in which Li_5_FeO_4_ is present. CO_2_ adsorption on the surface begins at 300 °C, and above 500 °C, CO_2_ capture was favored by the activation of intercrystalline diffusion. The maximum CO_2_ capture for both materials was achieved at around 700–715 °C. From this point, the investigation continues only with the material obtained from a 7:1 Li:Fe molar ratio, which was labeled as slag-Li_5_FeO_4_.

**Figure 5:**
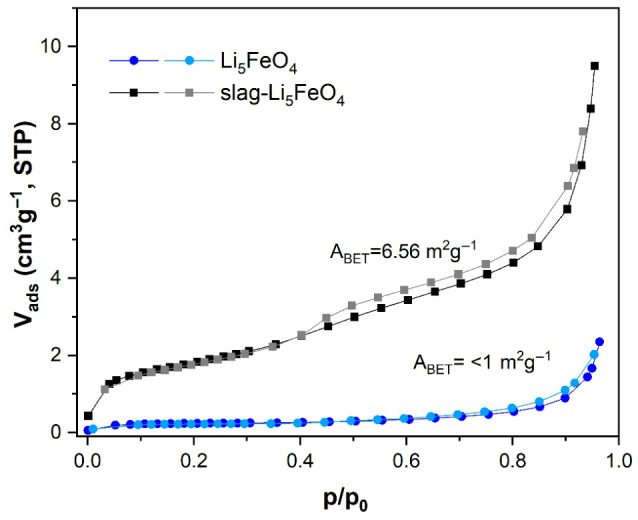
N_2_ adsorption–desorption isotherm of Li_5_FeO_4_ and slag-Li_5_FeO_4_.

**Figure 6:**
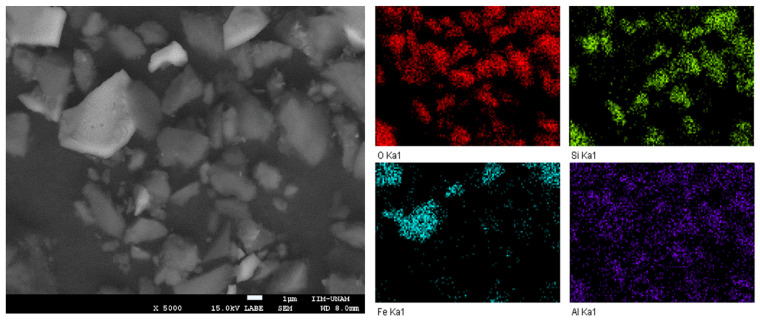
Scanning electron image and EDS elemental mapping of slag-Li_5_FeO_4_.

Lithium ferrite characterization continued with N_2_ adsorption–desorption isotherms and electron microscopy. According to the IUPAC classification, the isotherms are of type II, which indicates nonporous solids (Figure 5) [38]. The surface areas determined by the BET method were <1 and 6.56 m^2^ g^−1^ for Li_5_FeO_4_ (reagent-grade) and slag-Li_5_FeO_4_, respectively. The LABE image of slag-Li_5_FeO_4_ (Figure 6) shows polyhedral particles of around 1–7 μm. The EDS mapping indicates that Si and Fe are distributed within specific particles, corroborating the presence of Li_4_SiO_4_ and Li_5_FeO_4_. Moreover, the results show a highly homogeneous distribution of Al.

### 3.3. CO_2_ Sorption

**Figure 7:**
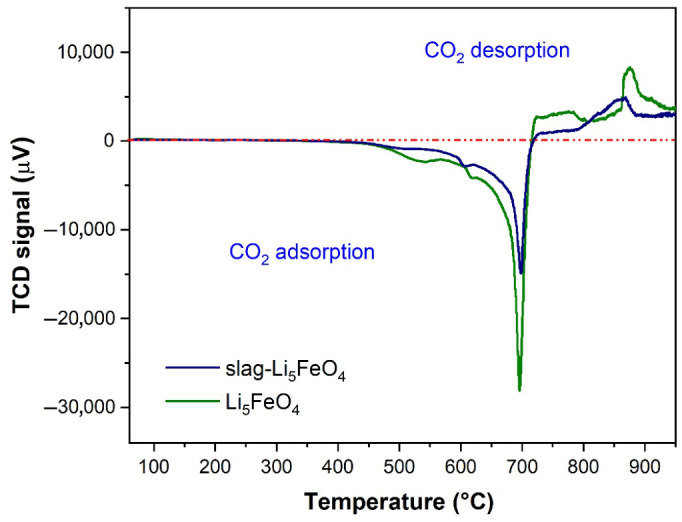
Temperature-programmed sorption–desorption tests of Li_5_FeO_4_ and slag-Li_5_FeO_4_.

The study of sorption behavior begins with temperature-programmed experiments, which allow the determination of the CO_2_ sorption–desorption capacity and the inversion temperature, as illustrated in Figure 7. CO_2_ sorption begins above 400 °C for both ferrite samples. Two peaks were observed in the capture process; the first was assigned to CO_2_ adsorption on the surface, and the second, and most important (700 °C, with a shoulder at 617 °C), was related to CO_2_ adsorption in the bulk of the material controlled by diffusive processes. Subsequently, at 716 °C, the equilibrium shifted towards desorption, which was not complete.

**Figure 8:**
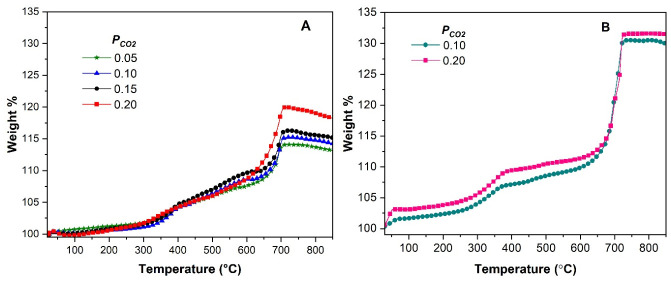
Dynamic TG curves of slag-Li_5_FeO_4_ (**A**) and Li_5_FeO_4_ (**B**) with different *P*_CO2_.

Dynamic TG experiments of slag-Li_5_FeO_4_ were carried out with different *P*_CO2_ as shown in Figure 8A. As reported for other lithium ceramics [4,5,33], two important temperature zones were identified, between 300 and around 580 °C and from 600 to 710 °C. Firstly, surface CO_2_ capture occurred, and an external shell of Li_2_CO_3_ and other secondary phases of calcium, aluminum and iron formed, covering the Li_5_FeO_4_ particles. Then, the increase in temperature activated different diffusion processes: intercrystalline diffusion of Li^+^ and O^2−^, and CO_2_ diffusion. Therefore, the bulk CO_2_ capture corresponds to the second weight increment. The decrease in *P*_CO2_ did not affect surface capture, but the activation of intercrystalline diffusion shifted to higher temperatures, and the final CO_2_ capture diminished. The temperature of the maximum CO_2_ sorption was around 710 °C (20 wt% with *P_CO_*_2_ = 0.20), as previously reported for lithium ferrites [6,11,36]. At higher temperatures, CO_2_ desorption begins slowly. For comparison, the thermograms of reagent-grade Li_5_FeO_4_ with *P*_CO2_ of 0.1 and 0.2 are shown in Figure 8B. The same behavior was observed, achieving a maximum CO_2_ capture of 31.5 wt% at 725 °C and no CO_2_ desorption. Notably, the decrease in *P*_CO2_ did not produce a significant change in CO_2_ sorption; the main difference was in superficial CO_2_ sorption, which was slightly less with a *P*_CO2_ of 0.1.

**Figure 9:**
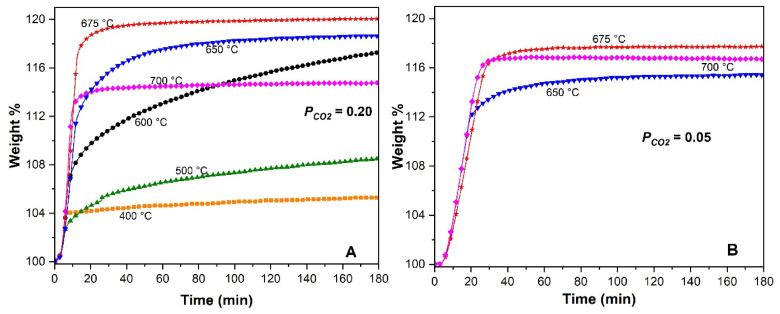
Isothermal analyses of slag-Li_5_FeO_4_ materials at different temperatures using *P_CO_*_2_: 0.2 (**A**) and 0.05 (**B**).

Figure 9 shows the effect of temperature and *P*_CO2_ on the CO_2_ sorption rate of the slag-Li_5_FeO_4_ material. The isothermal CO_2_ capture experiments were performed between 400 and 700 °C, with *P_CO_*_2_ of 0.20 and 0.05. As expected, all isotherms displayed exponential behavior. CO_2_ sorption increased with increasing temperature up to 675 °C and decreased at T ≥ 700 °C. At 400 °C, equilibrium was reached quickly, but CO_2_ sorption was low, only 5 wt%. With the increasing temperature, CO_2_ sorption improved, but equilibrium was not easily reached, as observed between 500 and 650 °C. This behavior improves as the temperature increases to 675 °C, reaching a maximum CO_2_ sorption of 20 wt%. Then, at 700 °C, CO_2_ sorption decreased due to desorption. As shown in Figure 9B, the process was slower at *P_CO_*_2_ of 0.05, but CO_2_ capture was only slightly reduced (17.8 wt% at 675 °C). In the first 20–30 min, curves at 650 and 700 °C showed a similar trend, while the curve at 675 °C was slightly slower. At this low CO_2_ partial pressure, the sorption–desorption equilibrium occurred at these close temperatures; as the temperature increased from 650 °C to 675 °C, CO_2_ capture increased, but the rate was slow because the adsorption–desorption equilibrium on the surface material was establishing. Then, when the temperature increased to 700 °C, the kinetic performance improved, and the CO_2_ sorption rate increased slightly again, although in the end, CO_2_ capture was not increased (16.8 wt%).

**Figure 10:**
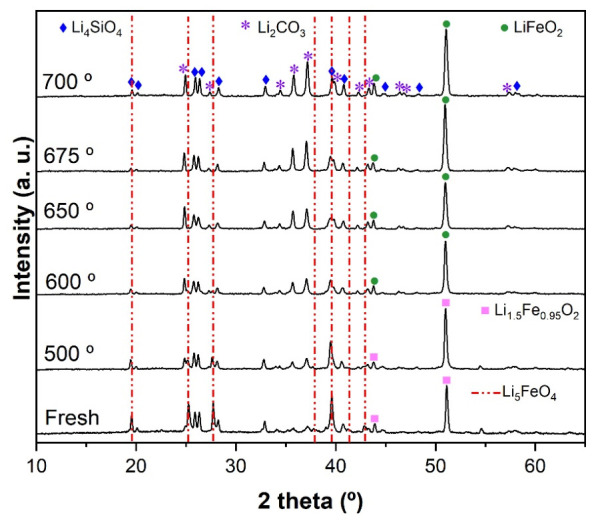
XRD patterns of slag-Li_5_FeO_4_ before and after CO_2_ capture (with *P_CO_*_2_ = 0.20) at different temperatures. Red dotted lines correspond to Li_5_FeO_4_ (PDF #00-037-1151).

DRX patterns of slag-ferrite after CO_2_ sorption at different temperatures are presented in Figure 10. According to reaction 1, Li_5_FeO_4_ reacts with CO_2_ to form Li_2_CO_3_ and LiFeO_2._ As shown, the intensity of the Li_5_FeO_4_ (red dotted lines, 00-037-1151 PDF file) and Li_4_SiO_4_ (01-076-1085 PDF file) signals decreased as the temperature of CO_2_ sorption increased, but at 700 °C, the intensity of Li_4_SiO_4_ increased slightly again; this is related to the CO_2_ desorption observed in the isothermal analyses at T > 675 °C. Li_2_CO_3_ (01-087-0729 PDF file), the principal product of the reaction, was observed at all CO_2_ sorption temperatures and obviously increased with the temperature. It is interesting to note that LiFeO_2_ was observed, corroborating that it does not react with CO_2_ at high temperatures, as confirmed by the absence of Fe_2_O_3_ (reaction 2).

**Figure 11:**
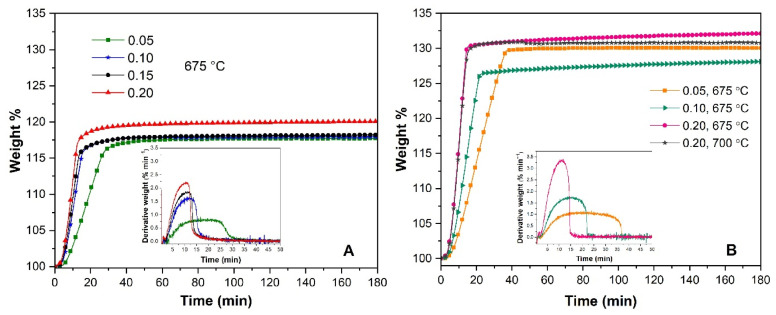
Isothermal analyses of slag-Li_5_FeO_4_ (**A**) and Li_5_FeO_4_ (**B**) at 675 °C with different *P_CO_*_2_. Inset: 1st derivative of weight % for the first minutes.

Figure 11A shows the isothermal CO_2_ capture behavior of slag-Li_5_FeO_4,_ performed at 675 °C with *P_CO_*_2_ ranging from 0.05 to 0.20. In the first 10 min, reducing the *P_CO_*_2_ to 0.15 and 0.10 had little effect on the sorption rate, but the effect was more pronounced when the *P_CO_*_2_ decreased to 0.05, corresponding to the slowest process (inset of Figure 11). However, after 3 h of the experiment, the maximum CO_2_ capture was 20 wt% with *P*_CO2_ = 0.20 and 18 wt% with *P*_CO2_ = 0.10, 0.15 or 0.05. As in the slag-Li_5_FeO_4_, Li_5_FeO_4_ displayed a slower CO_2_ sorption when the *P_CO_*_2_ decreased to 0.10 and 0.05 (Figure 11B). The maximum CO_2_ capture was 32 wt% at 675 °C with a *P_CO_*_2_ of 0.20. Furthermore, it is important to note that, with a *P_CO_*_2_ = 0.20, equilibrium was achieved at the same time (15 min) for both ferrites (with slag and reagent-grade). However, with a *P_CO_*_2_ less than 0.20, the CO_2_ sorption process was slower for reagent-grade ferrite than for slag-ferrite.

The kinetic analysis of the experimental data was performed with the Avrami–Erofeev model. The adsorption data were plotted as a straight line, ln(−ln(1 − *α*)) vs. ln *t*, with intercept ln*k* and slope *n* [35], where α is the degree of conversion (the ratio between the CO_2_ adsorption capacity at time *t* and the theoretical CO_2_ adsorption); *k* and *n* are the kinetic constant and the kinetic parameter, respectively. The magnitude of *n* gives the reaction rate, which is controlled by the formation and growth rate of reaction product crystals when *n* > 1. If *n* is approximately 0.5, the reaction proceeds under diffusion control [39]. Table 2 and Table 3 show the kinetic parameters *n* and *K* obtained under different temperature and *P*_CO2_ conditions. According to the *R^2^* value, the experimental data fit the model well, especially for the first stage of the process. For both ferrites, the constant *n* is greater than 1 for the first stage, suggesting that the reaction is controlled by the formation and growth of the carbonatation products [33], i.e., Li_2_CO_3_ and LiFeO_2_ crystals, according to reaction 1 and DRX results. During the second stage of the model, all *n* values are less than 1 across all experimental conditions, indicating that the reaction is controlled by CO_2_ and ion (Li^+^ and O^2−^) diffusion [35]. Furthermore, the *K* values are of the same order of magnitude for both materials, with the rapid reaction stage higher than the diffusion-controlled stage. This agrees with the literature and indicates that diffusion processes are the limiting step of the entire CO_2_ sorption process [5,18,20,22,35]. It is important to note that a decrease in *P_CO_*_2_ reduces *K* in both stages. The effect of temperature on the slag-ferrite is more pronounced in the second stage, where increasing *T* from 400 to 500 °C results in a significant increase in the *K* values, which remain constant up to 675 °C, after which they decrease in the same proportion. This behavior could be associated with a more pronounced sorption–desorption equilibrium at T ≥ 675 and low CO_2_ concentrations.

## 4. Conclusions

In the CCU field of research, the development of materials for CO_2_ capture and in situ conversion into value-added products has recently attracted attention. Furthermore, waste management and the circular economy are effective strategies for reducing the gap between technical and economic feasibility. In this context, this study demonstrates that lithium ferrite obtained from copper slag is an efficient material for CO_2_ capture at low CO_2_ partial pressures.

Li_5_FeO_4_ and Li_4_SiO_4_ were the main crystalline phases obtained. CO_2_ sorption increased with increasing temperature up to 675 °C, and decreased at T ≥ 700 °C, the temperature at which CO_2_ desorption begins. Slag-ferrite achieved a maximum CO_2_ capture of 20 wt% at 675 °C (*P_CO_*_2_ = 0.2), equivalent to 62.5% of the CO_2_ sorption of reagent-grade ferrite (32 wt%). The kinetic analysis using the Avrami–Erofeev model confirmed a two-step mechanism, with diffusion as the rate-limiting step in the overall sorption process. These findings provide valuable insights into the use of metallurgical slag to produce cost-effective materials for CCU applications, particularly for high-temperature CO_2_ capture. Finally, significant improvements in the material regeneration process are needed to further study its use in in situ CO_2_ conversion.

## Figures and Tables

**Table 1:** Chemical Composition of Copper slag by X-ray Fluorescence (XRF).

**Major elements** **(%)**	**Fe_2_O_3_**	**SiO_2_**	**Al_2_O_3_**	**SO_3_**	**K_2_O**	**CaO**	**TiO_2_**	**Na_2_O**	**MgO**	**P_2_O_5_**	**MnO**	**LOI**
	59.66	26.52	4.25	1.44	1.13	0.46	0.33	0.32	0.30	0.08	0.04	5.54
**Trace elements (ppm)**	**Cr**	**Ni**	**Cu**	**Zn**	**As**	**Mo**	**Pb**	**Zr**	**Co**	**Sb**	**Ba**	
	318	84	17,000	15,900	913	5140	1230	125	33	873	846	

LOI: loss of ignition.

**Table 2:** Kinetic Parameters of the Avrami–Erofeev Model for Slag-Li_5_FeO_4_.

T (°C)	*P_CO_*_2_	Rapid Reaction Stage			Diffusion Control Stage		
		*n*	*K* (s^−1^)	*R* ^2^	*n*	*K* (s^−1^)	*R* ^2^
400	0.20	2.5960	8.89 × 10^−4^	0.9892	0.1056	2.29 × 10^−14^	0.9759
500	0.20	2.4426	8.43 × 10^−4^	0.9912	0.3015	2.39 × 10^−7^	0.9840
600	0.20	2.7725	1.01 × 10^−3^	0.9958	0.3090	3.57 × 10^−6^	0.9976
650	0.20	2.5524	8.37 × 10^−4^	0.9954	0.1500	2.72 × 10^−7^	0.8740
675	0.20	2.5768	9.43 × 10^−4^	0.9948	0.0394	8.88 × 10^−14^	0.8003
675	0.15	2.6904	8.79 × 10^−4^	0.9924	0.0404	7.94 × 10^−15^	0.7346
675	0.10	2.7056	7.87 × 10^−4^	0.9880	0.0288	3.18 × 10^−19^	0.7564
675	0.05	2.4125	4.4 × 10^−4^	0.9672	0.0337	2.84 × 10^−17^	0.6424
700	0.20	2.8856	1.11 × 10^−3^	0.9948	0.0320	6.58 × 10^−21^	0.8278

**Table 3:** Kinetic Parameters of the Avrami–Erofeev Model for Li_5_FeO_4_.

T (°C)	*P_CO_*_2_	Rapid Reaction Stage			Diffusion Control Stage		
		*n*	*K* (s^−1^)	*R* ^2^	*n*	*K* (s^−1^)	*R* ^2^
675	0.20	2.7500	1.08 × 10^−3^	0.9960	0.0368	6.43 × 10^−7^	0.9972
675	0.10	2.5758	7.18 × 10^−4^	0.9830	0.0432	1.3 × 10^−8^	0.9946
675	0.05	2.5020	4.81 × 10^−4^	0.9322	0.0075	7.2 × 10^−21^	0.6228
700	0.20	2.8244	1.06 × 10^−3^	0.9925	0.0064	2.7 × 10^−21^	0.9772

## Data Availability

The datasets generated for this study are available on request to the corresponding author.
